# Ureter and bladder malakoplakia: a case report

**DOI:** 10.1093/bjrcr/uaag003

**Published:** 2026-01-19

**Authors:** Yu-Ping Ma, Jian-Guo Zhu, Qian-Ye Yong, Qiong Wang, Hai-Ge Li

**Affiliations:** Department of Radiology, The Second Affiliated Hospital of Nanjing Medical University, Nanjing, Jiangsu 210011, China; Department of Radiology, The Second Affiliated Hospital of Nanjing Medical University, Nanjing, Jiangsu 210011, China; Department of Radiology, The Second Affiliated Hospital of Nanjing Medical University, Nanjing, Jiangsu 210011, China; Department of Radiology, The Second Affiliated Hospital of Nanjing Medical University, Nanjing, Jiangsu 210011, China; Department of Radiology, The Second Affiliated Hospital of Nanjing Medical University, Nanjing, Jiangsu 210011, China

**Keywords:** ureteral and bladder disease, malakoplakia, inflammation, CTU, MR imaging

## Abstract

Malakoplakia (MP) is a rare granulomatous inflammation that primarily affects the urinary system and has a characteristic histological appearance. We report a case of ureteral and bladder MP associated with renal failure that clinically mimicked urothelial carcinoma. A 60-year-old woman presented to our clinic with a one-year history of left flank pain. Computed tomography urography (CTU) and magnetic resonance (MR) imaging revealed asymmetric thickening of the left ureteral and bladder walls, along with polypoid, contrast-enhancing intraluminal masses. The diagnosis was confirmed by histopathological evaluation, which demonstrated the pathognomonic Michaelis–Gutmann bodies.

## Introduction

Malakoplakia (MP) is a rare type of granulomatous inflammatory disease of unknown aetiology.^1^ It can affect any organ, but most commonly involves the mucosal surface of the urinary bladder.^2^ MP occurs more commonly in middle-aged women with chronic gram-negative bacterial infections (mostly with *E. coli*) and obstruction.^1^ Patients with urinary MP commonly present with a mass, fever, flank pain, hematuria, and pyuria. This report discusses the computed tomography urography (CTU) and magnetic resonance (MR) imagings of a case involving the ureter and bladder.

## Case presentation

A 60-year-old woman with a history of diabetes mellitus presented with left flank pain, low-grade fever, and dysuria of one year’s duration. Notably, she had undergone a left ureteroplasty for left ureteral stricture with unknown aetiology 30 years prior.

Urinalysis showed an elevated white blood cell count (2524/µL) and microscopic hematuria (37/µL). Serum creatinine (86.3 µmol/L, 0.98 mg/dL) and uric acid (566 µmol/L, 9.51 mg/dL) levels were elevated. No definitive tumour cells were identified on urine cytology.

**Figure 1.**
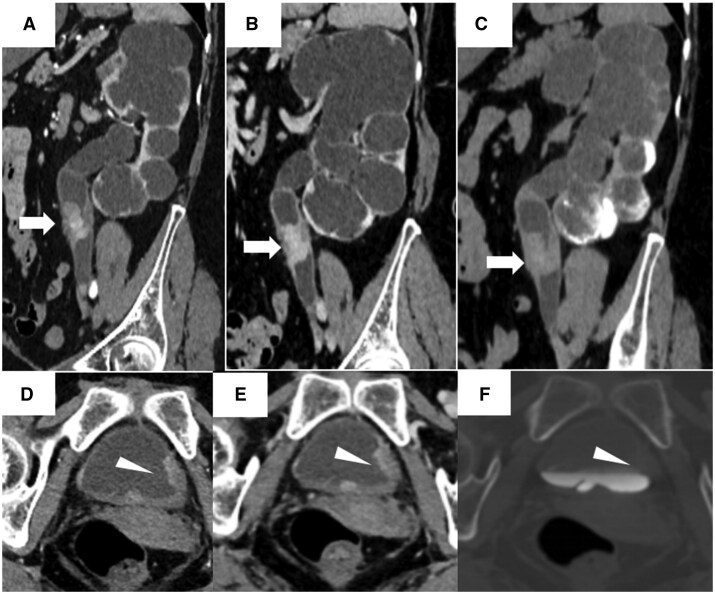
CT urography of ureteral and bladder malakoplakia. (A, D) Arterial phase; (B, E) venous phase; (C, F) excretory phase. (A-C) Axial images reveal annular thickening of the left upper ureter (arrow) with a polypoid, enhancing intraluminal mass, causing significant left-sided hydronephrosis. (D-F) Axial images demonstrate asymmetric thickening of the left posterior bladder wall (arrowhead) with marked mucosal enhancement.

Computed tomography urography demonstrated annular thickening of the upper segment of the left ureter with a polypoid, enhancing intraluminal mass, causing significant left hydronephrosis (Figure 1). The mass exhibited marked heterogeneous enhancement, with CT values of 81 HU, 107 HU, 92 HU, and 72 HU in the arterial, venous, delayed, and excretory phases, respectively. There was stranding involving the perireticular fat. Asymmetric thickening of the left posterior bladder wall was also noted, with significant mucosal enhancement (Figure 1).

**Figure 2.**
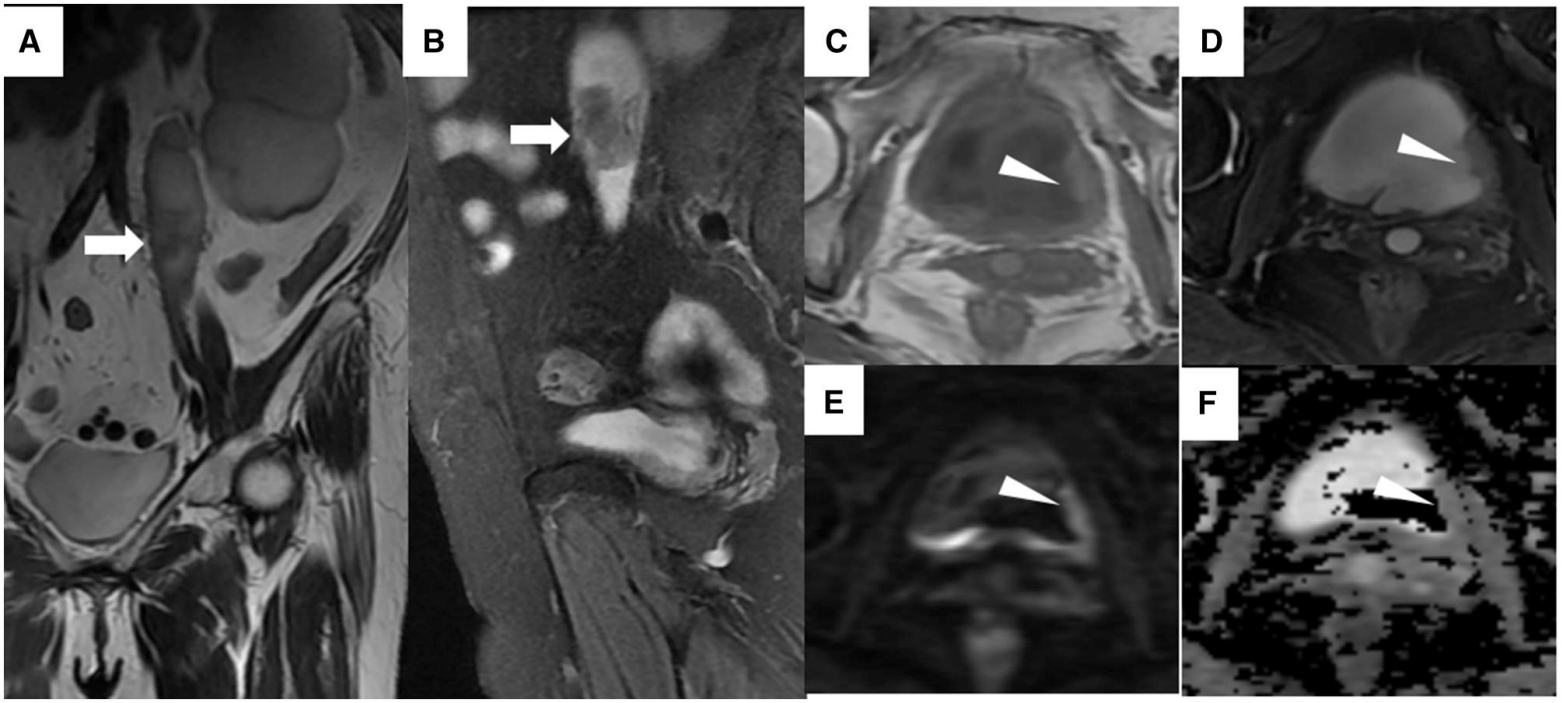
Magnetic resonance imaging of ureteral and bladder malakoplakia. (A) Coronal T2-weighted and (B) sagittal fat-saturated T2-weighted images demonstrate a hypointense intraluminal mass (arrow) in the left proximal ureter. (C) Axial T1-weighted and (D) axial fat-saturated T2-weighted images reveal asymmetric thickening of the left posterior bladder wall (arrowhead). (E) Axial diffusion-weighted image and (F) the corresponding apparent diffusion coefficient (ADC) map show mild restricted diffusion within the thickened wall.

Unenhanced magnetic resonance imaging was performed. The left ureter and bladder mass exhibited shorter T1 and T2 relaxation times compared to urine. (Figure 2). The mass measured approximately 3.5 cm × 2.2 cm. The left posterior bladder wall was slightly thickened, showing restricted diffusion with an apparent diffusion coefficient (ADC) value of approximately 1.6 × 10^−6^ mm^2^/s (Figure 2). No enlarged lymph nodes were observed in the abdominal and pelvic cavity.

**Figure 3.**
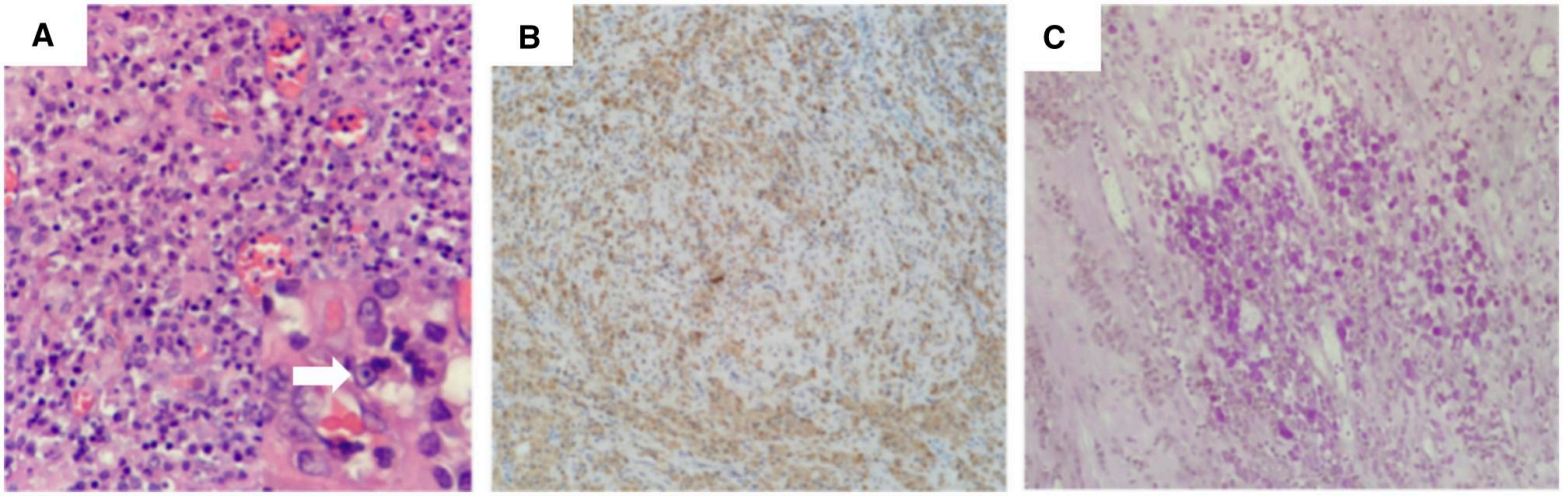
Histopathological confirmation of malakoplakia. (A) Photomicrograph (H&E stain, ×200) reveals sheets of histiocytes containing eosinophilic cytoplasm and pathognomonic Michaelis–Gutmann bodies (inset, arrow). (B) Immunohistochemical staining confirms CD68 positivity in the histiocytes (×100). (C) Periodic acid–Schiff (PAS) staining highlights the Michaelis–Gutmann bodies and cytoplasm in purplish-red (×100).

Given the indeterminate nature of the left ureteral and bladder masses with extensive involvement, significant left hydronephrosis, and deteriorating renal function, the patient underwent left nephroureterectomy, radical cystectomy and bilateral ureteral cutaneous ureterostomy. Postoperative pathological examination revealed granulomatous lesions containing round, basophilic intracytoplasmic inclusions known as Michaelis–Gutmann bodies (Figure 3). Periodic acid-Schiff (PAS) staining was positive, confirming the diagnosis of MP. At the two weeks follow-up, the patient was feeling well and reported resolution of her flank pain. At the 3-month follow-up, the patient was asymptomatic, and laboratory exams showed that the value of inflammatory markers had decreased.

## Discussion

MP is a rare chronic granulomatous inflammatory disease of unknown aetiology. It most commonly involves the mucosal surface of the urinary bladder.^2^ Less common sites of involvement include the kidneys (affecting 15% of patients), gastrointestinal tract, lungs, genital tract, and skin.^3–5^ In the present case, the left ureter and bladder were primarily affected.

Various Gram-negative organisms have been implicated in MP, most commonly Escherichia coli.^6^ Risk factors include immunocompromised states, such as organ transplantation, HIV infection, or diabetes mellitus (as in our patient).^7^ MP has a peak incidence in middle age, with a reported female-to-male ratio of 4:1, likely reflecting the higher incidence of urinary tract infections in females.^2^ Patients with ureteral and bladder MP commonly present with intraluminal masses, fever, flank pain, hematuria, and pyuria.

Radiologically, MP in the ureter and bladder can manifest as solitary or multiple polypoid nodules or diffuse wall thickening, often associated with obstruction or vesicoureteral reflux. These nodules range in size from a few millimetres to several centimetres. On non-contrast scans, the lesions typically exhibit soft-tissue density/signal intensity. Following contrast administration, they typically demonstrate moderate to marked heterogeneous enhancement. On ultrasonography, renal malakoplakia typically presents as hypoechoic, poorly defined cortical masses or diffuse parenchymal thickening, with or without associated hydronephrosis.^6^ Regarding FDG-PET/CT, malakoplakia is known to demonstrate significant FDG avidity due to the dense inflammatory infiltrate of activated macrophages and associated bacteria has a high glycolytic rate.^8^ This intense avidity creates a critical diagnostic challenge, as malakoplakia can be indistinguishable from an aggressive renal malignancy or lymphoma on FDG-PET. Therefore, in a patient with a relevant history of recurrent urinary tract infections (particularly with E. coli), the constellation of a ‘tumour-mimicking’ lesion on CT/MR/US and intense FDG avidity should prompt consideration of malakoplakia.

Due to its nonspecific imaging and clinical presentation, pathological examination is essential for a definitive diagnosis. Microscopically, MP is characterized by chronic granulomatous inflammation with abundant foamy histiocytes containing eosinophilic granular cytoplasm. The nodular and heterogeneously enhancing masses of the ureter and bladder seen on CT/MRI directly correspond to the inflammatory and granulomatous process observed under the microscope. The histiocytic infiltration accounts for the soft tissue density and mass-like appearance. The pathognomonic feature is the presence of Michaelis–Gutmann bodies, which appear as basophilic, concentric, owl-eye-like inclusions within some histiocytes.^2^ These bodies are positive on PAS, calcium, and iron stains. A limitation of this case is that chemical shift MRI was not performed; therefore, the presence of microscopic fat associated with foamy macrophages could not be assessed.

First-line treatment involves long-term antibiotics with good intracellular penetration, such as fluoroquinolones and rifampin. Surgical intervention is generally reserved for cases refractory to antibiotic therapy.^6^

## Learning points

- MP is a rare chronic granulomatous inflammatory disease that primarily involves the urinary system, and often affects middle-aged females.
- Radiologically, MP can manifest as polypoid nodules or diffuse wall thickening, often associated with obstruction or vesicoureteral reflux.
- Its imaging and clinical manifestations are nonspecific. Histopathological examination is necessary for a definitive diagnosis, with Michaelis–Gutmann bodies being the pathognomonic feature.
